# *Araucaria angustifolia* chloroplast genome sequence and its relation to other Araucariaceae

**DOI:** 10.1590/1678-4685-GMB-2018-0213

**Published:** 2019-11-14

**Authors:** José Henrique S. G. Brandão, Nureyev F. Rodrigues, Maria Eguiluz, Frank Guzman, Rogerio Margis

**Affiliations:** 1 PPGBM, Departamento de Genética, Universidade Federal do Rio Grande do Sul, (UFRGS), Porto Alegre, RS, Brazil.; 2 PPGBCM, Centro de Biotecnologia, Universidade Federal do Rio Grande do Sul (UFRGS), Porto Alegre, RS, Brazil.; 3 Departamento de Biofísica, Universidade Federal do Rio Grande do Sul (UFRGS), Porto Alegre, RS, Brazil.

**Keywords:** Brazilian pine, plastid, genome, cpDNA, conservation

## Abstract

*Araucaria angustifolia* is endemic to southern Brazil. Known as Brazilian pine, *A. angustifolia* is the only native conifer species with economic and social relevance in this country. Due to massive exploitation, it has suffered a significant population decline and currently is classified as critically endangered. This encouraged the scientific community to investigate genetic features in Brazilian pine to increase resources for management and preservation. In this work, RNA-Seq data was used to determine the complete nucleotide sequence of the *A. angustifolia* chloroplast genome (cpDNA). The cpDNA is 146,203 bp in length and contains 122 genes, including 80 protein-coding genes, 5 ribosomal RNA genes, and 37 tRNA genes. Coding regions comprise 45.02%, 4.96% correspond to rRNAs and tRNAs, and 50.02% of the genome encompasses non-coding regions. Genes found in the inverted repeat (IR) are present as single copy, with exception of the *rrn5* and *trn*I-CAU loci. The typical LSC, SSC, IRa and IRb organization reported in several land-plant groups is not present in *A. angustifolia* cpDNA. Phylogenetic analyses using Bayesian and Maximum Likelihood methods clustered *A. angustifolia* in the Araucariaceae family, with *A. heterophylla* and *A. columnaris* as congeneric species*.* The screening of *A. angustifolia* cpDNA reveled 100 SSRs, 14 of them corresponding to tetrapolymer loci.

*Araucaria angustifolia* (Bertol.) Kuntze syn. *Columbea angustifolia*, also known as Brazilian pine, is a native Brazilian tree of the class Pinopsida, order Pinales, and family Araucariaceae. It is widely distributed in the southern and southeastern areas of Brazil but also occurs in limited areas from Argentina and Paraguay (de Souza *et al.*, 2009). *A. angustifolia* is a dioecious wind-pollinated species with a mixed seed dispersion by barochoric authocory and birds (Lowe *et al.*, 2018). The seeds maintain elevated levels of water and active metabolic rates at the mature stage, resulting in a rapid loss of viability (Astarita *et al.*, 2004). Due to seed recalcitrance to storage, conservation strategies are restricted mainly to propagation by embryogenic cultures (Steiner *et al.*, 2008).

*A. angustifolia* is one of the most important trees in its region of natural occurrence due to its relevant ecological, economic, and social functions. Its seeds are rich in starch, proteins, and flavonoids, having a high nutritional value during the winter season. As results of its social and economic relevance, *A. angustifolia* went through an indiscriminate exploitation and a substantial population decline, having been categorized as a critically endangered species in the International Union for the Conservation of Nature and Natural Resources (IUCN), Red List of Threatened Species (Thomas, 2013). The taxonomic classification of the genus *Araucaria* is well resolved (Stefenon *et al.*, 2006). It comprises 19 species with an interesting distribution worldwide. The species are distributed only in tropical and subtropical zones of the Southern hemisphere (Stefenon *et al.*, 2006). Seventeen species are present in Oceania, 13 of which are endemic to the small archipelago New Caledonia (Lu *et al.*, 2014). The two remaining species, *Araucaria araucana,* and *Araucaria angustifolia,* are present in southern South America (Lu *et al.*, 2014).

Brazilian pine has been targeted by genetic studies that mainly focused on somatic embryogenesis, with the purpose of developing technologies for the conservation and genetic improvement of this species. One of these studies has generated RNA-seq data from early stage tissues and the libraries are available in the NCBI database (Elbl *et al.*, 2015). Once high-throughput sequencing data is generated, it can be used in a plethora of ways beyond the original purpose, and relevant information can be further explored from the targeted organism. In the present study, the RNA-seq data composed by 24 libraries (Elbl *et al.*, 2015) was used as input to perform the complete assembly and annotation of the *A. angustifolia* chloroplast (cp) genome. The paired-end sequence reads were filtered against 58 Pinidae cp genomes (Table S1) using BWA software with two mismatches allowed (Li and Durbin, 2009). The reads were used for an assembly *de novo* with ABySS software (Simpson *et al.*, 2009). The cp genome scaffolds were orientated using cp genome sequences of *Araucaria heterophylla* (NC_026450.1) using BLASTN (Camacho *et al.*, 2009). A gap region relative to an intergenic region was filled in after Sanger sequencing using the primers F: 5’ ACCGTGAGGGTTCAAGTCC and R: 3’ GTGGCACG AGGATTTTCAGT. For this purpose, total DNA was extracted by the CTAB method from young leaves of an *A. angustifolia* tree. DNA quality was evaluated by electrophoresis in a 1% agarose gel, and quantity was determined using a NanoDrop spectrophotometer (NanoDrop Technologies, Wilmington, DE, USA). Genes were annotated using GeSeq (Tillich *et al.*, 2017) and BLAST similarity searches. Transfer RNAs (tRNAs) were predicted using the Aragorn program (Laslett and Canback, 2004) implemented in the GeSeq program and confirmed by comparison with the appropriate homologs in *A. heterophylla*. The circular cp genome map was drawn using the online program OGDRAW (Lohse *et al.*, 2013).

To determine the phylogenetic relationships of *A. angustifolia* in the Pinidae division and corroborate with the Brazilian pine plastid genome sequence, a set of 73 cp protein-coding sequences (Table S2) from 18 conifer species, 17 belonging to Pinidae (Table S3) and *Ginkgo biloba* serving as outgroup were used. Nucleotide sequences were aligned separately using MUSCLE available in MEGA version 6.0 (Tamura *et al.*, 2013). Alignments were concatenated and nucleotide positions of each gene were specified and a Bayesian tree was generated using MrBayes version 3.2.6 (Ronquist et *al.*, 2012), with the JC evolutionary model as determined by MODELTEST version 3.7 (Posada and Crandall, 1998), and 10,000,000 generations sampled every 100 generations. The first 25% of trees were discarded as burn-in to produce a consensus phylogram, with posterior probability (PP) values for each node. Maximum Likelihood (ML) analysis was also applied, using RaxML (Stamatakis, 2014) program and the ML tree was compared to Bayesian topology. The phylogenetic trees were rooted and visualized using FigTree software (http://tree.bio.ed.ac.uk/software/figtree/).

Simple sequence repeats (SSRs) were detected using MISA perl script, available at (http://pgrc.ipk-gatersleben.de/misa/), with thresholds of 10 repeat units for mononucleotide SSRs, five repeat units for di- and trinucleotide SSRs, and three repeat units for tetra-, penta- and hexanucleotide SSRs. The interruption threshold among SSRs, which indicates the maximum difference between two SSRs was 50 base pairs. A total of 229,914,266 high quality Illumina paired-end reads from the *A. angustifolia* transcriptome libraries generated via the HiScanSQ platform and available at NCBI Sequence Read Archive (SRA) under accession number SRP039545 were filtered against Pinidae cp genomes. The 2,107,993 obtained reads were *de novo* assembled into non-redundant contigs and singletons covering about 99.65% of the cp genome (minimum coverage of 23 reads, maximum coverage of 1,780 reads). Two final large scaffolds were obtained and joined into a single cp circular genome after the use of Sanger sequencing. The complete cp genome of *A. angustifolia* is 146,203 bp in size and was submitted to GenBank (accession number: MH599004). This size is similar to those found in other *Araucaria* species (Ruhsam *et al.*, 2015).

In *A. angustifolia*, duplicated genes present in inverted repeat regions IRa and IRb are found as a single copy, with exception of a sequence of 513 bp corresponding to the *rrn5* gene, placed between *clpP* and *psbB*, suggesting a recombination event, and the two inverted copies from *tRNA-CAU* gene (Figure 1). The same pattern was described in other species of the Araucariaceae family, *Agathis dammara* and *Wollemia nobilis*, which lack canonical IRs and harbor double inverted copies of *rrn5* and *tRNA-CAU* in their plastomes (Wu and Chaw, 2014; Yap *et al.*, 2015). A reduced size of IRa and IRb was described in *Pinus taeda* L. (Asaf *et al.*, 2018). Loss of the IR was also reported in the chloroplast genomes of some species of Pinaceae and Cupressophytes (Wu *et al.*, 2011).[Bibr B25]

**Figure 1:**
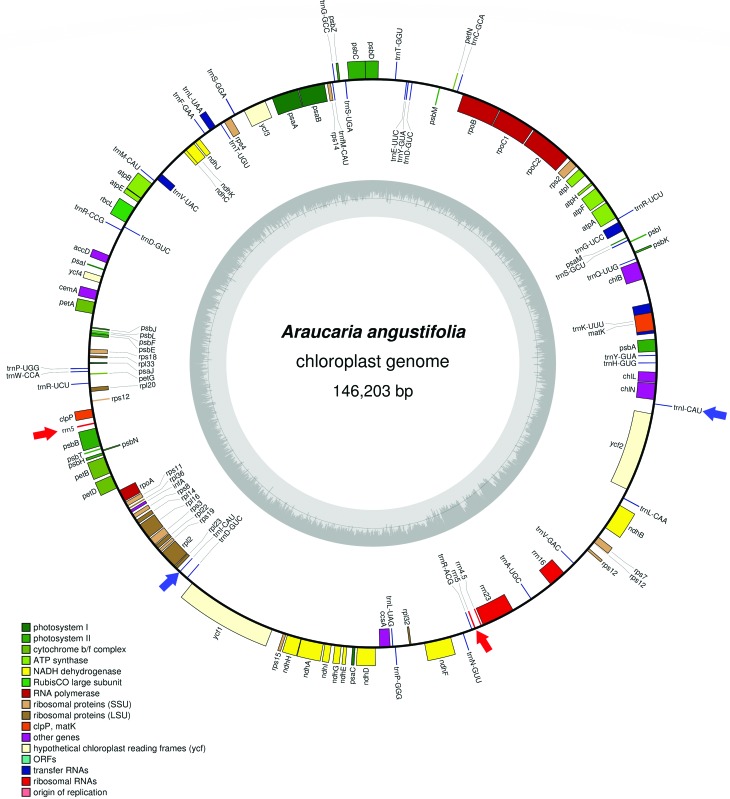
Gene map of the *Araucaria angustifolia* chloroplast genome. Genes drawn inside the circle are transcribed counterclockwise and those outsides are clockwise. Genes belonging to different functional groups are indicated by different tonalities. The darker gray in the inner circle corresponds to the GC content, while the lighter gray corresponds to the AT content. Red and blue arrows indicate, respectively, double inverted copies of the *rrn5* and *trn*I-CAU genes.

The coding sequences comprise 45.02%, 4.96% correspond to rRNAs and tRNAs, and 50.02% of the genome comprises non-coding regions, including introns, pseudogenes and intergenic spacers (Table 1). In general, all genomic features showed similarity in size, structure, and gene abundance with other *Araucaria* species (Table S4) (Ruhsam *et al.*, 2015). The genome contains 122 genes in total, which includes 120 single-copy genes corresponding to 80 protein-coding genes, 36 transfer RNA (tRNA) genes and four ribosomal genes (rRNA) (Figure 1, Table 1). The cp genome has 14 intron-containing genes: 9 protein-coding genes, one pseudogene, and four tRNA genes. The *rps12* gene*,* a trans-spliced gene entirely located in the LSC region, and the *ycf3* gene contain two introns each; the other genes have only one intron each. The *trnK-UUU* intron has 2,407 bp, with the largest intron encompassing also the *matK* gene, a common feature of land-plants chloroplast genomes.

**Table 1:** Summary of the *Araucaria angustifolia* chloroplast genome features.

Feature	*Araucaria angustifolia*
Total cpDNA size	146,203 bp
Protein coding regions (%)	45.02%
rRNA and tRNA (%)	4.96%
Introns (% total)	8.3%
Intergenic sequences and pseudogenes (%)	41.72%
Number of genes	122
Number of protein coding genes	80
Number of tRNA genes	36
Number of rRNA genes	4
Number of duplicated genes	2
Pseudogenes	1
GC content (%)	36.54%

A phylogenetic analysis was performed to evaluate the position of *A. angustifolia* in the Araucariaceae family and subclass Pinidae, and 73 protein-coding genes from other 18 conifers were used for this purpose. The final alignment reached 55,435 nucleotides. These species were intentionally sampled to comprise the main representative taxa of the subclass Pinidae, while *Ginkgo biloba* was used as outgroup. The Bayesian analysis resulted in a consistent phylogenetic relationship of *A. angustifolia* and the 18 conifer represented species (Figure 2). Within Pinidae we found, two branches, the first one represented by the family Pinaceae, order Pinales, and the other by the families Cupressaceae, Podocarpaceae, Araucariaceae comprising Conifer I and Conifer II, respectively (Lu *et al.*, 2014). Within Conifer II, the genera and species distribution is clear and well supported among Araucariaceae and Podocarpaceae, comprising the order Araucariales and Cupressaceae and comprising the order Cupressales (Lu *et al.*, 2014). Within Araucariaceae, *Araucaria angustifolia*, *Araucaria heterophylla,* and *Araucaria columnaris* were grouped together in the genus *Araucaria*, which grouped with *Agathis dammara* and *Wollemia nobilis*. In the monophyletic *Araucaria* clade, *Araucaria columnaris* and *Araucaria heterophylla*, representing endemic species from New Caledonia and Norfolk Island, Australia, respectively, are grouped together, and another basal branch corresponds to *Araucaria angustifolia*, endemic from South America (Lu *et al.*, 2014). The strongly supported topology within the genus *Araucaria*, family Araucariaceae, and among the other taxa (Cupressaceae, Podocarpaceae, and Pinaceae) is congruent with a series of phylogenetic studies (Lu *et al.*, 2014). The ML analysis corroborated the Bayesian approach (Figure S1), which strongly reinforces the importance of cpDNA for phylogenetic inference.

**Figure 2:**
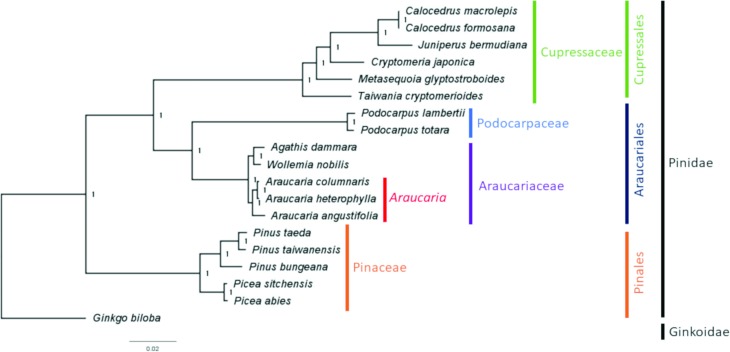
Phylogenetic tree of 18 species of Pinidae based on 73 cp protein-coding genes generated by Bayesian method. Numbers above each node are posterior probability values. Family and order are also indicated. *Ginkgo biloba* was used as outgroup.

Using the MISA perl script, 100 simple sequence repeats (SSRs) were detected in *A. angustifolia* cpDNA. The 53 homopolymers A/T and 24 dipolymers AT were the most common SSRs, while 14 different tetrapolymers and a single hexapolymer were also found (Table S5). SSR pentapolymers were not present in the cpDNA. The present *A. angustifolia* chloroplast genome is the first complete cpDNA sequence for this species and shows a set of features that could be further explored for population and phylogenetic studies within this group. Moreover, the present study increases the genetic and genomic resources available in *Araucaria* and shows that, as reported in bryophytes and angiosperms (Shi *et al.*, 2016), the plastome sequence can be straightforwardly assembled from transcriptome data generared for conifers.

## Supplementary Material

The following online material is available for this article:

Figure S1 Phylogenetic tree of 18 species of Pinidae based on 73 cp protein-coding genes generated using Maximum Likelihood.

Table S1 List of 58 Pinidae complete chloroplast genomes used for mapping plastid reads from *Araucaria angustifolia.*

Table S2 List of 73 chloroplast protein-coding genes used in the phylogenetic analysis.

Table S3 List of 18 plastome sequences of Pinidae included in the Bayesian and Maximum Likelihood phylogenetic analysis.

Table S4 Comparison of cpDNA characteristics in different species from Araucariaceae

Table S5 List of simple sequence repeats (SSRs) identified in the *Araucaria angustifolia* chloroplast genome.
