# Supplementary material for: Araucaria angustifolia chloroplast genome sequence and its relation to other Araucariaceae
Source: Genet Mol Biol. 2019 Nov 14;42(3):671–6. doi: 10.1590/1678-4685-GMB-2018-0213 (PMC6905450; doi:10.1590/1678-4685-GMB-2018-0213)
Supplement: Supplementary file 1 [file 1415-4757-GMB-42-3-2018-0213-20190902-suppl2.pdf]

Supplementary Material to "*Araucaria angustifolia* chloroplast genome sequence and its relation to other Araucariaceae"

Table S1 - List of 58 Pinidae complete chloroplast genomes used in chloroplast genome assembling of *Araucaria angustifolia*

No.	Taxon	GenBank accession number	Study
1	*Abies koreana*	KP742350.1	(Yi *et al.*, 2016b)
2	*Abies nephrolepis*	KT834974.1	(Yi *et al.*, 2016a)
3	*Agathis dammara*	AB830884.1	(Wu and Chaw 2014)
4	*Amentotaxus argotaenia*	KR780582.1	(Li *et al.*, 2015a)
5	*Calocedrus formosana*	AB831010.1	(Wu and Chaw, 2014)
6	*Cathaya argyrophylla*	AB547400.1	(Lin *et al.*, 2010)
7	*Cedrus deodara*	NC_014575.1	(Lin *et al.*, 2010)
8	*Cephalotaxus oliveri*	KC136217.1	(Yi *et al.*, 2013)
9	*Cryptomeria japônica*	AP009377.1	(Hirao *et al.*, 2008)
10	*Cunninghamia lanceolata*	KC427270.1	-
11	*Cupressus gigantea*	KT315754.1	(Li *et al.*, 2016a)
12	*Glyptostrobus pensilis*	KU302768.1	(Hao *et al.*, 2016)
13	*Juniperus bermudiana*	KF866297.1	(Guo *et al.*, 2014)
14	*Juniperus cedrus*	KT378453.1	(Guo *et al.*, 2016)
15	*Juniperus monosperma*	KF866298.1	(Guo *et al.*, 2014)
16	*Juniperus scopulorum*	KF866299.1	(Guo *et al.*, 2014)
17	*Juniperus virginiana*	KF866300.1	(Guo *et al.*, 2014)
18	*Keteleeria davidiana*	NC_011930.1	(Wu *et al.*, 2009)
19	*Larix decídua*	AB501189.1	(Wu *et al.*, 2011)
20	*Metasequoia glyptostroboides*	KR061358.1	(Chen *et al.*, 2015)
21	*Nageia nagi*	AB830885.1	(Wu and Chaw, 2014)
22	*Picea abies*	HF937082.1	(Nystedt *et al.*, 2013)
23	*Picea glauca*	KT634228.1	(Jackman *et al.*, 2015)
24	*Picea jezoensis*	KT337318.1	(Yang *et al.*, 2016)
25	*Picea morrisonicola*	AB480556.1	(Wu *et al.*, 2011)
26	*Picea sitchensis*	EU998739.3	(Cronn *et al.*, 2008)
27	*Picea sitchensis*	KU215903.2	(Coombe *et al.*, 2016)
28	*Pinus armandii*	KP412541.1	(Li *et al.*, 2015b)
29	*Pinus bungeana*	KR873010.1	(Li *et al.*, 2015c)
30	*Pinus contorta*	EU998740.4	(Cronn *et al.*, 2008)
31	*Pinus fenzeliana* var. *dabeshanensis*	KX255674.1	(Duan *et al.*, 2016)
32	*Pinus gerardiana*	EU998741.4	(Cronn *et al.*, 2008)
33	*Pinus koraiensis*	AY228468.2	-
34	*Pinus krempfii*	EU998742.4	(Cronn *et al.*, 2008)
35	*Pinus lambertiana*	EU998743.4	(Cronn *et al.*, 2008)
36	*Pinus longaeva*	EU998744.3	(Cronn *et al.*, 2008)
37	*Pinus massoniana*	KC427272.1	-
38	*Pinus monophylla*	EU998745.4	(Cronn *et al.*, 2008)
39	*Pinus nelsonii*	EU998746.4	(Cronn *et al.*, 2008)
40	*Pinus sibirica*	KT723438.2	-
41	*Pinus tabuliformis*	KT740995.1	(Yu *et al.*, 2017)
42	*Pinus taeda*	KY964286.1	(Asaf *et al.*, 2018)

No.	Taxon	GenBank accession number	Study
43	*Pinus taiwanensis*	KP771703.1	(Fang *et al.*, 2015)
44	*Pinus thunbergii*	D17510.1	(Tsudzuki *et al.*, 1992)
45	*Pinus thunbergii*	NC_001631.1	(Wakasugi *et al.*, 1994)
46	*Podocarpus lambertii*	KJ010812.1	(Vieira *et al.*, 2014)
47	*Podocarpus totara*	KC306742.1	-
48	*Pseudolarix amabilis*	LC095867.1	(Sudianto *et al.*, 2016)
49	*Pseudotsuga sinensis* var. *wilsoniana*	AB601120.1	(Wu *et al.*, 2011)
50	*Retrophyllum piresii*	KJ617081.1	(do Nascimento Vieira *et al.*, 2016)
51	*Sciadopitys verticillata*	KT601210.1	(Li *et al.*, 2016b)
52	*Sequoia sempervirens*	KR075871.1	-
53	*Taiwania flousiana*	KC427274.1	-
54	*Taxus mairei*	KJ123824.1	(Zhang *et al.*, 2014)
55	*Taxus wallichiana* var. *chinensis*	KX431996.1	(Jia and Liu, 2017)
56	*Torreya fargesii*	KT027377.1	(Tao *et al.*, 2016)
57	*Tsuga chinensis*	LC095866.1	(Sudianto *et al.*, 2016)
58	*Wollemia nobilis*	KP259800.1	(Yap *et al.*, 2015)

References from data presented in Table S1

Asaf S, Khan AL, Khan MA, Shahzad R, Lubna, Kang SM, Al-Harrasi A, Al-Rawahi A and Lee I-J (2018) Complete chloroplast genome sequence and comparative analysis of loblolly pine (*Pinus taeda* L.) with related species. PLoS One 13:e0192966.

Chen J, Hao Z, Xu H, Yang L, Liu G, Sheng Y, Zheng C, Zheng W, Cheng T and Shi J (2015) The complete chloroplast genome sequence of the relict woody plant *Metasequoia glyptostroboides* Hu et Cheng. Front Plant Sci 6:1–11.

Coombe L, Warren RL, Jackman SD, Yang C, Vandervalk BP, Moore RA, Pleasance S, Coope RJ, Bohlmann J, Holt RA *et al.* (2016) Assembly of the complete Sitka Spruce chloroplast genome using 10X Genomics' GemCode sequencing data. PLoS One 11:e0163059.

Cronn R, Liston A, Parks M, Gernandt DS, Shen R and Mockler T (2008) Multiplex sequencing of plant chloroplast genomes using Solexa sequencing-by-synthesis technology. Nucleic Acids Res 36:e122.

do Nascimento VL, Rogalski M, Faoro H, Pacheco de FFH, Goulart dos AK, Assine PGF, Onofre NR, de Oliveira PF, Maltempi de SE and Pedro GM (2016) The plastome sequence of the endemic Amazonian conifer, *Retrophyllum piresii* (Silba) C.N.Page, reveals different recombination events and plastome isoforms. Tree Genet Genomes 12:10.

Duan RY, Yang LM, Lv T, Wu GL and Huang MY (2016) The complete chloroplast genome sequence of *Pinus dabeshanensis*. Conserv Genet Resour 8:395–397.

Fang MF, Wang YJ, Zu YM, Dong WL, Wang RN, Deng TT and Li ZH (2015) The complete chloroplast genome of the Taiwan red pine *Pinus taiwanensis* (Pinaceae). Mitochondrial DNA 27:1–2.

Guo Q, Bianba D and Zheng W (2016) Characterization of the complete chloroplast genome of *Juniperus cedrus* (Cupressaceae). Mitochondrial DNA Part A, DNA mapping, Seq Anal 27:4355–4356.

Guo W, Grewe F, Cobo-Clark A, Fan W, Duan Z, Adams RP, Schwarzbach AE and Mower JP (2014) Predominant and substoichiometric isomers of the plastid genome coexist within Juniperus plants and have shifted multiple times during cupressophyte evolution. Genome Biol Evol 6:580–90.

Hao Z, Cheng T, Zheng R, Xu H, Zhou Y, Li M, Lu F, Dong Y, Liu X, Chen J *et al.* (2016) The complete chloroplast genome sequence of a relict conifer *Glyptostrobus pensilis*: Comparative analysis and insights into dynamics of chloroplast genome rearrangement in Cupressophytes and Pinaceae. PLoS One 11:e0161809.

Hirao T, Watanabe A, Kurita M, Kondo T and Takata K (2008) Complete nucleotide sequence of the *Cryptomeria japonica* D. Don. chloroplast genome and comparative chloroplast genomics: diversified genomic structure of coniferous species. BMC Plant Biol 8:70.

Jackman SD, Warren RL, Gibb EA, Vandervalk BP, Mohamadi H, Chu J, Raymond A, Pleasance S, Coope R, Wildung MR *et al.* (2015) Organellar genomes of White Spruce (*Picea glauca*): Assembly and annotation. Genome Biol Evol 8:29–41.

Jia XM and Liu XP (2017) Characterization of the complete chloroplast genome of the Chinese yew *Taxus chinensis* (Taxaceae), an endangered and medicinally important tree species in China. Conserv Genet Resour 9:197–199.

Li H, Guo Q and Zheng W (2016a) The complete chloroplast genome of *Cupressus gigantea*, an endemic conifer species to Qinghai-

Tibetan Plateau. Mitochondrial DNA Part A, DNA mapping, Seq Anal 27:3743–3744.

Li J, Gao L, Chen S, Tao K, Su Y and Wang T (2016b) Evolution of short inverted repeat in cupressophytes, transfer of accD to nucleus in *Sciadopitys verticillata* and phylogenetic position of Sciadopityaceae. Sci Rep 6:20934.

Li J, Gao L, Tao K, Su Y and Wang T (2015a) The complete chloroplast genome sequence of *Amentotaxus argotaenia* (Taxaceae). Mitochondrial DNA 1–2.

Li ZH, Qian ZQ, Liu ZL, Deng TT, Zu YM, Zhao P and Zhao GF (2015b) The complete chloroplast genome of Armand pine *Pinus armandii*, an endemic conifer tree species to China. Mitochondrial DNA 27:1–2.

Li ZH, Zhu J, Yang YX, Yang J, He JW and Zhao GF (2015c) The complete plastid genome of Bunge's pine *Pinus bungeana* (Pinaceae). Mitochondrial DNA 27:1–2.

Lin CP, Huang JP, Wu CS, Hsu CY and Chaw SM (2010) Comparative chloroplast genomics reveals the evolution of Pinaceae genera and subfamilies. Genome Biol Evol 2:504–517.

Nystedt B, Street NR, Wetterbom A, Zuccolo A, Lin YC, Scofield DG, Vezzi F, Delhomme N, Giacomello S, Alexeyenko A *et al.* (2013) The Norway spruce genome sequence and conifer genome evolution. Nature 497:579–84.

Sudianto E, Wu CS, Lin CP and Chaw SM (2016) Revisiting the plastid phylogenomics of Pinaceae with two complete plastomes of Pseudolarix and Tsuga. Genome Biol Evol 8:1804–1811.

Tao K, Gao L, Li J, Chen S, Su Y and Wang T (2016) The complete chloroplast genome of *Torreya fargesii* (Taxaceae). Mitochondrial DNA Part A 27:3512–3513.

Tsudzuki J, Nakashima K, Tsudzuki T, Hiratsuka J, Shibata M, Wakasugi T and Sugiura M (1992) Chloroplast DNA of black pine retains a residual inverted repeat lacking rRNA genes: Nucleotide sequences of trnQ, trnK, psbA, trnI and trnH and the absence of rps16. MGG Mol Gen Genet 232:206–214.

Vieira LDN, Faoro H, Rogalski M, Fraga HP de F, Cardoso RLA, de Souza EM, de Oliveira PF, Nodari RO and Guerra MP (2014) The complete chloroplast genome sequence of *Podocarpus lambertii*: Genome structure, evolutionary aspects, gene content and SSR detection. PLoS One 9:e90618.

Wakasugi T, Tsudzuki J, Ito S, Nakashima K, Tsudzuki T and Sugiura M (1994) Loss of all ndh genes as determined by sequencing the entire chloroplast genome of the black pine Pinus thunbergii. Proc Natl Acad Sci U S A 91:9794–9798.

Wu CS and Chaw SM (2014) Highly rearranged and size-variable chloroplast genomes in conifers II clade (cupressophytes): evolution towards shorter intergenic spacers. Plant Biotechnol J 12:344–353.

Wu CS, Lai YT, Lin CP, Wang YN and Chaw SM (2009) Evolution of reduced and compact chloroplast genomes (cpDNAs) in gnetophytes: selection toward a lower-cost strategy. Mol Phylogenet Evol 52:115–24.

Wu CS, Lin CP, Hsu CY, Wang RJ and Chaw SM (2011) Comparative chloroplast genomes of Pinaceae: Insights into the mechanism of diversified genomic organizations. Genome Biol Evol 3:309–319.

Yang JC, Joo M, So S, Yi DK, Shin CH, Lee YM and Choi K (2016) The complete plastid genome sequence of *Picea jezoensis* (Pinaceae: Piceoideae). Mitochondrial DNA Part A 27:3761–3763.

Yap JYS, Rohner T, Greenfield A, Van Der Merwe M, McPherson H, Glenn W, Kornfeld G, Marendy E, Pan AYH, Wilton A *et al.* (2015) Complete chloroplast genome of the Wollemi Pine (*Wollemia nobilis*): Structure and evolution. PLoS One 10:e0128126.

Yi DK, Choi K, Joo M, Yang JC, Mustafina FU, Han JS, Son DC, Chang KS, Shin CH and Lee YM (2016a) The complete chloroplast genome sequence of *Abies nephrolepis* (Pinaceae: Abietoideae). J Asia-Pacific Biodivers 9:245–249.

Yi DK, Yang JC, So S, Joo M, Kim DK, Shin CH, Lee YM and Choi K (2016b) The complete plastid genome sequence of *Abies koreana* (Pinaceae: Abietoideae). Mitochondrial DNA 27:2351–2353.

Yi X, Gao L, Wang B, Su YJ and Wang T (2013) The complete chloroplast genome sequence of *Cephalotaxus oliveri* (Cephalotaxaceae): evolutionary comparison of cephalotaxus chloroplast DNAs and insights into the loss of inverted repeat copies in gymnosperms. Genome Biol Evol 5:688–98.

Yu Z, Peng S and Yang P (2017) The complete chloroplast genome of the southern Chinese pine P*inus tabuliformis* (Pinales: Pinaceae). Mitochondrial DNA Part A 28:13–14.

Zhang Y, Ma J, Yang B, Li R, Zhu W, Sun L, Tian J and Zhang L (2014) The complete chloroplast genome sequence of *Taxus chinensis* var. mairei (Taxaceae): Loss of an inverted repeat region and comparative analysis with related species. Gene 540:201–9.
