# Supplementary material for: Araucaria angustifolia chloroplast genome sequence and its relation to other Araucariaceae
Source: Genet Mol Biol. 2019 Nov 14;42(3):671–6. doi: 10.1590/1678-4685-GMB-2018-0213 (PMC6905450; doi:10.1590/1678-4685-GMB-2018-0213)
Supplement: Supplementary file 2 [file 1415-4757-GMB-42-3-2018-0213-20190902-suppl3.pdf]

Supplementary Material to "*Araucaria angustifolia* chloroplast genome sequence and its relation to other Araucariaceae"

Table S2 - List of 73 chloroplast protein coding genes used in the phylogenetic analysis.

Gene	Name	Gene	Name
accD	acetyl-CoA carboxylase beta subunit	*psbE*	cytochrome b559 alpha subunit
atpA	ATP synthase CF1 alpha chain	*psbF*	photosystem II reaction center subunit VI
atpB	ATPase beta chain	*psbH*	photosystem II reaction center protein H
atpE	ATP synthase epsilon chain	*psbI*	photosystem II protein I
atpF	ATPase subunit I	*psbJ*	photosystem II reaction center subunit X
atpH	ATPase III subunit	*psbK*	photosystem II protein K
atpI	ATPase IV subunit	*psbL*	photosystem II protein L
ccsA	cytochrome c biogenesis protein	*psbM*	photosystem II protein M
cemA	heme-binding protein	*psbN*	photosystem II reaction center protein N
chlB	protochlorophyllide reductase 58kDa chain	*psbT*	photosystem II protein T
chlN	photochlorophyllide reductase subunit N	*psbZ*	photosystem II protein Z
infA	translation initiation factor 1	*rbcL*	ribulose-1,5-bisphosphate carboxylase/oxygenase large subunit
matK	maturase K	*rpl2*	ribosomal protein L2
ndhA	NADH dehydrogenase subunit A	*rpl14*	ribosomal protein L14
ndhB	NADH dehydrogenase subunit B	*rpl16*	ribosomal protein L16
ndhC	NADH dehydrogenase subunit C	*rpl20*	ribosomal protein L20
ndhD	NADH dehydrogenase subunit D	*rpl22*	ribosomal protein L22
ndhE	NADH dehydrogenase subunit E	*rpl23*	ribosomal protein L23
ndhF	NADH dehydrogenase subunit F	*rpl32*	ribosomal protein L32
ndhG	NADH dehydrogenase subunit G	*rpl36*	ribosomal protein L36
nhdH	NADH dehydrogenase subunit H	*rpoA*	RNA polymerase alpha chain
ndhI	NADH dehydrogenase 18 kDa subunit	*rpoB*	RNA polymerase beta chain
ndhJ	NADH dehydrogenase 30 kDa subunit	*rpoC1*	RNA polymerase beta subunit-1
ndhK	NADH dehydrogenase subunit K	*rpoC2*	RNA polymerase beta subunit-2
petA	apocytochrome f precursor	*rps2*	ribosomal protein S2
petB	cytochrome b6	*rps3*	ribosomal protein S3
petD	cytochrome b6/f complex subunit 4	*rps4*	ribosomal protein S4
petG	cytochrome b6/f complex subunit 5	*rps7*	ribosomal protein S7
petL	cytochrome b6/f complex subunit 6	*rps8*	ribosomal protein S8
petN	cytochrome b6/f complex subunit N	*rps11*	ribosomal protein S11
psaA	photosystem I P700 apoprotein A1	*rps12*	ribosomal protein S12
psaB	photosystem I P700 apoprotein A2	*rps14*	ribosomal protein S14
psaC	photosystem I iron-sulfur center	*rps15*	ribosomal protein S15
psbA	photosystem II protein D1	*rps19*	ribosomal protein S19
psbB	photosystem II P680 chlorophyll A apoprotein	*ycf3*	photosystem I assembly protein Ycf3
psbC	photosystem II 43 kDa protein	*ycf4*	photosystem I assembly protein Ycf4
psbD	photosystem II protein D2		
