# Supplementary material for: Araucaria angustifolia chloroplast genome sequence and its relation to other Araucariaceae
Source: Genet Mol Biol. 2019 Nov 14;42(3):671–6. doi: 10.1590/1678-4685-GMB-2018-0213 (PMC6905450; doi:10.1590/1678-4685-GMB-2018-0213)
Supplement: Supplementary file 3 [file 1415-4757-GMB-42-3-2018-0213-20190902-suppl4.pdf]

Supplementary Material to "*Araucaria angustifolia* chloroplast genome sequence and its relation to other Araucariaceae"

Table S3. List of 17 plastome sequences of conifers included in the phylogenetic analysis.

	Species	Family	Order	Acc	Study
**1**	*Agathis dammara*	Araucariaceae	Araucariales	NC_023119.1	(Wu and Chaw, 2014)
**2**	*Araucaria angustifolia*	Araucariaceae	Araucariales	MH599004	Present study
**3**	*Araucaria columnaris*	Araucariaceae	Araucariales	KM678417	(Hollingsworth *et al.*, 2009)
**4**	*Araucaria heterophylla*	Araucariaceae	Araucariales	NC_026450	(Ruhsam *et al.*, 2015)
**5**	*Wollemia nobilis*	Araucariaceae	Araucariales	NC_027235.1	(Yap *et al.*, 2015)
**6**	*Podocarpus lambertii*	Podocarpaceae	Araucariales	NC_023805	(Vieira *et al.*, 2014)
**7**	*Podocarpus totara*	Podocarpaceae	Araucariales	NC_020361	Direct Submission
**8**	*Calocedrus formosana*	Cupressaceae	Cupressales	KX832620.1	(Wu and Chaw, 2014)
**9**	*Calocedrus macrolepis*	Cupressaceae	Cupressales	KX832621	(Qu *et al.*, 2017)
**10**	*Cryptomeria japonica*	Cupressaceae	Cupressales	NC_010548	(Hirao *et al.*, 2008)
**11**	*Juniperus bermudiana*	Cupressaceae	Cupressales	NC_024021	(Guo *et al.*, 2014)
**12**	*Metasequoia glyptostroboides*	Cupressaceae	Cupressales	NC_027423	(Chen *et al.*, 2015)
**13**	*Taiwania cryptomerioides*	Cupressaceae	Cupressales	NC_016065	(Wu *et al.*, 2011)
**14**	*Ginkgo biloba*	Ginkgoaceae	Ginkgoales	MG922618.1	(Lin *et al.*, 2012)
**15**	*Picea abies*	Pinaceae	Pinales	NC_021456	(Nystedt *et al.*, 2013)
**16**	*Picea sitchensis*	Pinaceae	Pinales	KU215903.2	(Coombe *et al.*, 2016)
**17**	*Pinus bungeana*	Pinaceae	Pinales	NC_028421	(Li *et al.*, 2015)
**18**	*Pinus taeda*	Pinaceae	Pinales	KY964286	(Asaf *et al.*, 2018)
**19**	*Pinus taiwanensis*	Pinaceae	Pinales	NC_035069	(Fang *et al.*, 2015)

References

Asaf S, Khan AL, Khan MA, Shahzad R, Lubna, Kang SM, Al-Harrasi A, Al-Rawahi A and Lee I-J (2018) Complete chloroplast genome sequence and comparative analysis of loblolly pine (*Pinus taeda* L.) with related species. PLoS One 13:e0192966.

Chen J, Hao Z, Xu H, Yang L, Liu G, Sheng Y, Zheng C, Zheng W, Cheng T and Shi J (2015) The complete chloroplast genome sequence of the relict woody plant *Metasequoia glyptostroboide*s Hu et Cheng. Front Plant Sci 6:1–11.

Coombe L, Warren RL, Jackman SD, Yang C, Vandervalk BP, Moore RA, Pleasance S, Coope RJ, Bohlmann J, Holt RA *et al.* (2016) Assembly of the complete Sitka Spruce chloroplast genome using 10X genomics' GemCode sequencing data. PLoS One 11:e0163059.

Fang MF, Wang YJ, Zu YM, Dong WL, Wang RN, Deng TT and Li ZH (2015) The complete chloroplast genome of the Taiwan red pine *Pinus taiwanensis* (Pinaceae). Mitochondrial DNA 27:1–2.

Guo W, Grewe F, Cobo-Clark A, Fan W, Duan Z, Adams RP, Schwarzbach AE and Mower JP (2014) Predominant and substoichiometric isomers of the plastid genome coexist within Juniperus plants and have shifted multiple times during cupressophyte evolution. Genome Biol Evol 6:580–90.

Hirao T, Watanabe A, Kurita M, Kondo T and Takata K (2008) Complete nucleotide sequence of the *Cryptomeria japonica* D. Don. chloroplast genome and comparative chloroplast genomics: Diversified genomic structure of coniferous species. BMC Plant Biol 8:70.

Hollingsworth ML, Andra CA, Forrest LL, Richardson J, Pennington RT, Long DG, Cowan R, Chase MW, Gaudeul M and Hollingsworth PM (2009) Selecting barcoding loci for plants: Evaluation of seven candidate loci with species-level sampling in three divergent groups of land plants. Mol Ecol Resour 9:439–457.

Li ZH, Zhu J, Yang YX, Yang J, He JW and Zhao GF (2015) The complete plastid genome of Bunge's pine *Pinus bungeana* (Pinaceae). Mitochondrial DNA 27:1–2.

Lin CP, Wu CS, Huang YY and Chaw SM (2012) The complete chloroplast genome of *Ginkgo biloba* reveals the mechanism of inverted repeat contraction. Genome Biol Evol 4:374–381.

Nystedt B, Street NR, Wetterbom A, Zuccolo A, Lin YC, Scofield DG, Vezzi F, Delhomme N, Giacomello S, Alexeyenko A *et al.* (2013) The Norway spruce genome sequence and conifer genome evolution. Nature 497:579–84.

Qu XJ, Jin JJ, Chaw SM, Li DZ and Yi TS (2017) Multiple measures could alleviate long-branch attraction in phylogenomic reconstruction of Cupressoideae (Cupressaceae). Sci Rep 7:1–11.

Ruhsam M, Rai HS, Mathews S, Ross TG, Graham SW, Raubeson LA, Mei W, Thomas PI, Gardner MF, Ennos RA *et al.* (2015) Does complete plastid genome sequencing improve species discrimination and phylogenetic resolution in Araucaria? Mol Ecol Resour 15:1067–1078.

Vieira LDN, Faoro H, Rogalski M, Fraga HP de F, Cardoso RLA, de Souza EM, de Oliveira PF, Nodari RO and Guerra MP (2014) The complete chloroplast genome sequence of *Podocarpus lambertii*: genome structure, evolutionary aspects, gene content and SSR detection. PLoS One 9:e90618.

Wu CS and Chaw SM (2014) Highly rearranged and size-variable chloroplast genomes in conifers II clade (cupressophytes): evolution towards shorter intergenic spacers. Plant Biotechnol J 12:344–353.

Wu CS, Wang YN, Hsu CY, Lin CP and Chaw SM (2011) Loss of different inverted repeat copies from the chloroplast genomes of pinaceae and cupressophytes and influence of heterotachy on the evaluation of gymnosperm phylogeny. Genome Biol Evol 3:1284–1295.

Yap JYS, Rohner T, Greenfield A, Van Der Merwe M, McPherson H, Glenn W, Kornfeld G, Marendy E, Pan AYH, Wilton A *et al.* (2015) Complete chloroplast genome of the Wollemi Pine (*Wollemia nobilis*): Structure and evolution. PLoS One 10:e0128126.
