# Supplementary material for: Araucaria angustifolia chloroplast genome sequence and its relation to other Araucariaceae
Source: Genet Mol Biol. 2019 Nov 14;42(3):671–6. doi: 10.1590/1678-4685-GMB-2018-0213 (PMC6905450; doi:10.1590/1678-4685-GMB-2018-0213)
Supplement: Supplementary file 4 [file 1415-4757-GMB-42-3-2018-0213-20190902-suppl5.pdf]

Supplementary Material to "*Araucaria angustifolia* chloroplast genome sequence and its relation to other Araucariaceae"

Table S4. Comparison of cpDNA characteristics in different species of Araucariaceae.

**Characteristics**	***Araucaria***			***Wollemia***	***Agathis***
	A. angustifolia	***A. columnaris***	***A. heterophylla***	***W. nobilis***	***A. dammara***
GenBank Accession no.	MH599004[a]	KM678417[b]	NC_026450.1[b]	NC_027235.1[b]	NC_023119.1[b]
Size (bp)	146,203	146,799	146,723	145,63	145,625
GC content (%)	36.54	36.6	36.7	36.5	36.54
Total number of genes	122	124	124	122	123
Total number of unique genes	120	119	121	118	119
Protein-coding genes	80	82	82	82	82
Ribosomal RNAs	5	5	5	5	5
Transfer RNAs	37	37	37	35	36

[a]Present study.
[b]References are provided in Table S3.
