# Supplementary material for: Araucaria angustifolia chloroplast genome sequence and its relation to other Araucariaceae
Source: Genet Mol Biol. 2019 Nov 14;42(3):671–6. doi: 10.1590/1678-4685-GMB-2018-0213 (PMC6905450; doi:10.1590/1678-4685-GMB-2018-0213)
Supplement: Supplementary file 5 [file 1415-4757-GMB-42-3-2018-0213-20190902-suppl1.pdf]

Supplementary Material to "*Araucaria angustifolia* chloroplast genome sequence and its relation to other Araucariaceae"

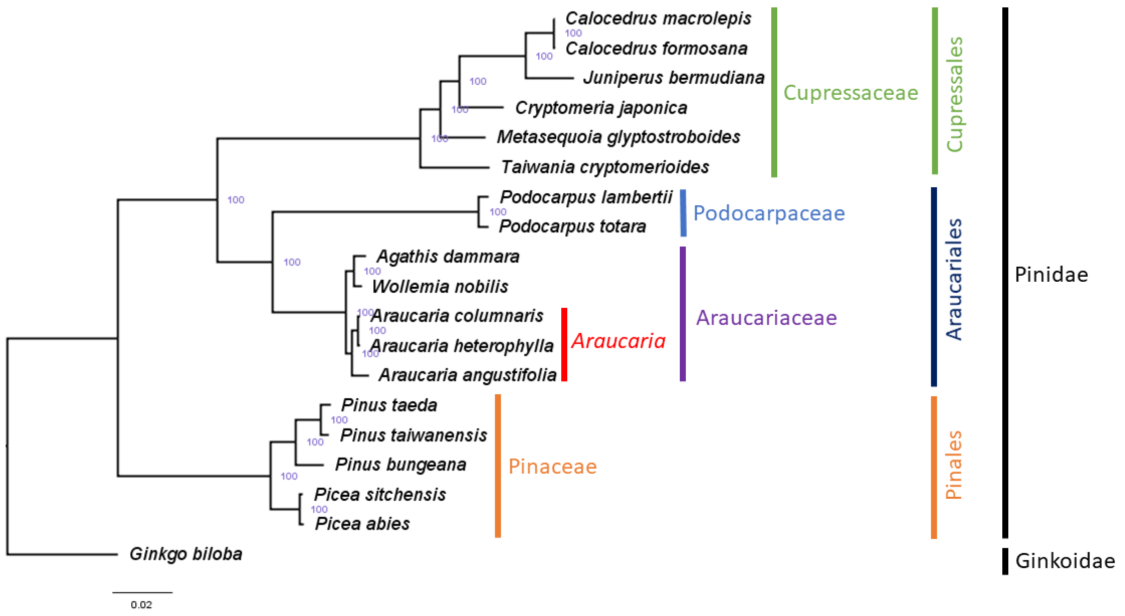

Figure S1 - Phylogenetic tree of 18 species of Pinidae based on 73 cp protein-coding genes generated using Maximum Likelihood. Numbers above each node indicate bootstrap values. Family and order are also indicated. *Ginkgo biloba* was used as outgroup.
