# Supplementary material for: Araucaria angustifolia chloroplast genome sequence and its relation to other Araucariaceae
Source: Genet Mol Biol. 2019 Nov 14;42(3):671–6. doi: 10.1590/1678-4685-GMB-2018-0213 (PMC6905450; doi:10.1590/1678-4685-GMB-2018-0213)
Supplement: Supplementary file 6 [file 1415-4757-GMB-42-3-2018-0213-20190902-suppl6.pdf]

Supplementary Material to "*Araucaria angustifolia* chloroplast genome sequence and its relation to other Araucariaceae"

Table S5. List of simple sequence repeats identified in the *Araucaria angustifolia* chloroplast genome.

**SSR sequence**	**Number of repeats**																	**Total**
	**3**	**4**	**5**	**6**	**7**	**8**	**9**	**10**	**11**	**12**	**13**	**14**	**15**	**16**	**17**	**18**	**19**	
**A/T**	-	-	-	-	-	-	-	12	13	8	8	4	4	2		1	1	53
**C/G**	-	-	-	-	-	-	-	1	-	-	-	-	-	-	-	-	-	1
**AG/CT**	-	-	2	1	1	-	-	-	-	-	-	-	-	-	-	-	-	4
**AT/AT**	-	-	4	4	3	5	3	3	1	-	1	-	-	-	-	-	-	24
**AAG/CTT**	-	-	1	-	1	-	-	-	-	-	-	-	-	-	-	-	-	2
**AAT/ATT**	-	-	1	-	-	-	-	-	-	-	-	-	-	-	-	-	-	1
**AAAC/GTTT**	1	-	-	-	-	-	-	-	-	-	-	-	-	-	-	-	-	1
**AAAG/CTTT**	5	-	-	-	-	-	-	-	-	-	-	-	-	-	-	-	-	5
**AAAT/ATTT**	1	1	-	-	-	-	-	-	-	-	-	-	-	-	-	-	-	2
**AACC/GGTT**	1	-	-	-	-	-	-	-	-	-	-	-	-	-	-	-	-	1
**ACCT/AGGT**	1	-	-	-	-	-	-	-	-	-	-	-	-	-	-	-	-	1
**AGAT/ATCT**	4	-	-	-	-	-	-	-	-	-	-	-	-	-	-	-	-	4
**AGATCT/AGATCT**	1	-	-	-	-	-	-	-	-	-	-	-	-	-	-	-	-	1
**Total**																		**100**
